# Supplementary figures and images for: Pro-Osteogenic Properties of Violina pumpkin (Cucurbita moschata) Leaf Extracts: Data from In Vitro Human Primary Cell Cultures
Source: Nutrients. 2021 Jul 30;13(8):2633. doi: 10.3390/nu13082633 (PMC8399764; doi:10.3390/nu13082633)

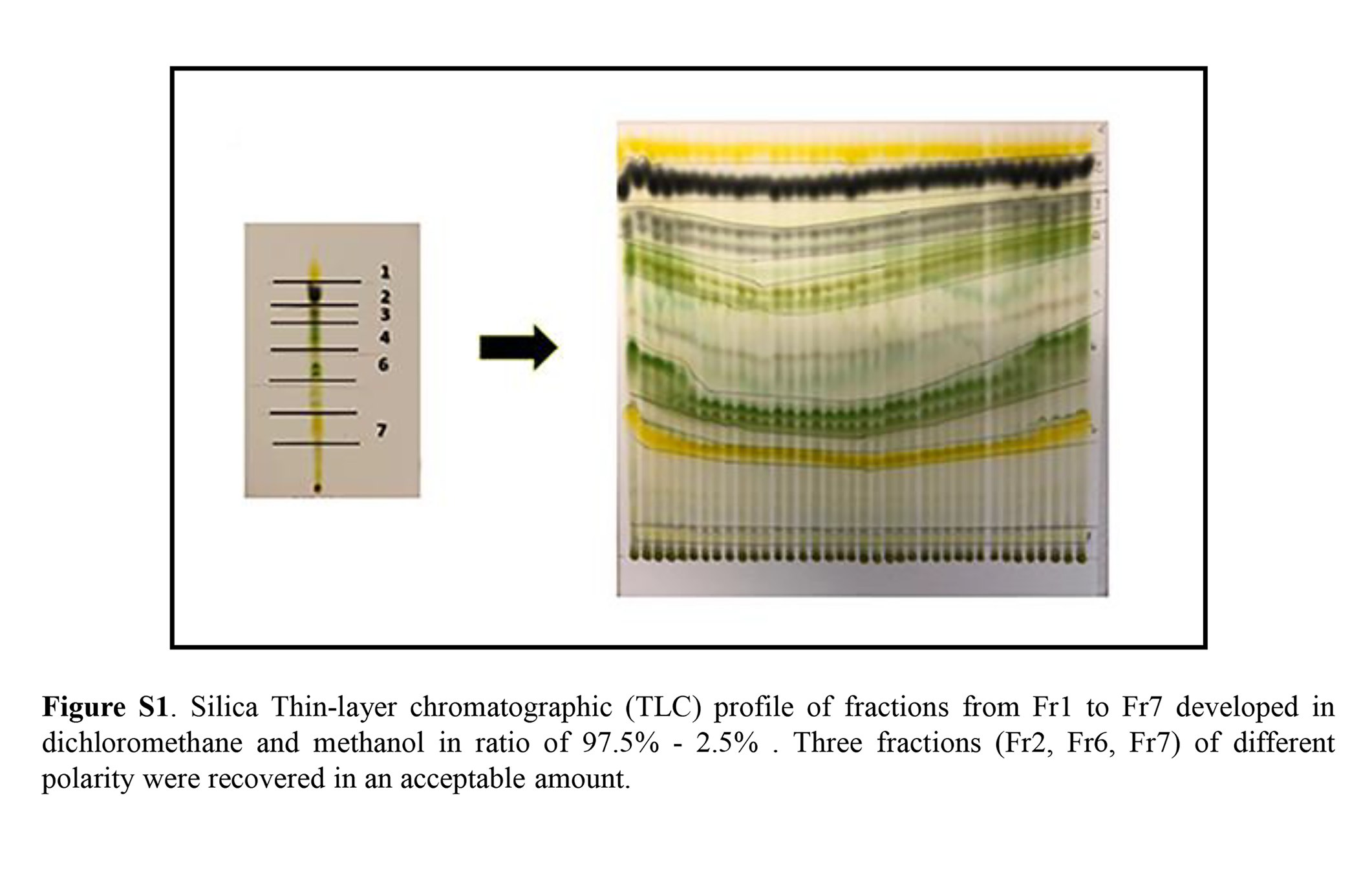

Supplement: Supplementary file 1 [file nutrients-13-02633-s001.zip › Figure S1.tif]

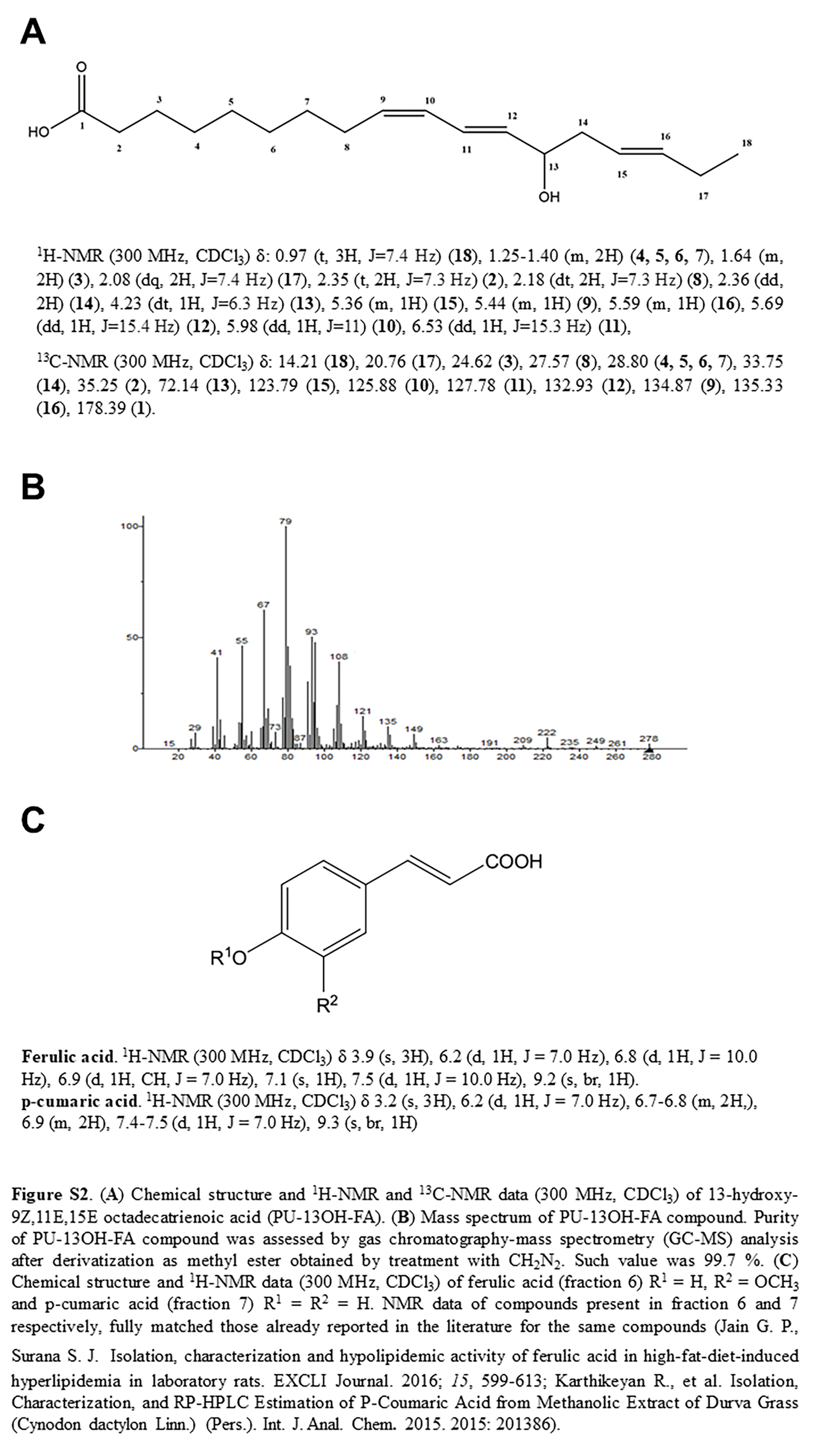

Supplement: Supplementary file 1 [file nutrients-13-02633-s001.zip › Figure S2.tif]
